# Vaccination With Recombinant Filamentous *fd* Phages Against Parasite Infection Requires TLR9 Expression

**DOI:** 10.3389/fimmu.2018.01173

**Published:** 2018-05-29

**Authors:** João F. Gomes-Neto, Rossella Sartorius, Fábio B. Canto, Thamyres S. Almeida, André A. Dias, Carlos-Henrique D. Barbosa, Guilherme A. Melo, Ana Carolina Oliveira, Pedro-Henrique N. Aguiar, Carlos R. Machado, Herbert L. de Matos Guedes, Marcelo F. Santiago, Alberto Nóbrega, Piergiuseppe De Berardinis, Maria Bellio

**Affiliations:** ^1^Instituto de Microbiologia Paulo de Góes, Universidade Federal do Rio de Janeiro, Rio de Janeiro, Brazil; ^2^Institute of Protein Biochemistry, CNR, Naples, Italy; ^3^Instituto de Biofísica Carlos Chagas Filho, Universidade Federal do Rio de Janeiro (UFRJ), Rio de Janeiro, Brazil; ^4^Instituto de Ciências Biológicas, Universidade Federal de Minas Gerais (UFMG), Belo Horizonte, Brazil; ^5^Instituto de Biofísica Carlos Chagas Filho, Polo Xerém, Universidade Federal do Rio de Janeiro (UFRJ), Rio de Janeiro, Brazil; ^6^National Institute for Vaccine Development and Technology (INCTV), CNPq-MCT, Belo Horizonte, Brazil

**Keywords:** *fd* phages, *Tlr9*, *Trypanosoma cruzi*, cytotoxic T cell, delivery system, vaccine, CD8 T cells

## Abstract

Recombinant filamentous *fd* bacteriophages (r*fd*) expressing antigenic peptides were shown to induce cell-mediated immune responses in the absence of added adjuvant, being a promising delivery system for vaccination. Here, we tested the capacity of r*fd* phages to protect against infection with the human protozoan *Trypanosoma cruzi*, the etiologic agent of Chagas Disease. For this, C57BL/6 (B6) and *Tlr9^−^*^/^*^−^* mice were vaccinated with r*fd* phages expressing the OVA_257–264_ peptide or the *T. cruzi*-immunodominant peptides PA8 and TSKB20 and challenged with either the *T. cruzi* Y-OVA or Y-strain, respectively. We found that vaccination with r*fd* phages induces anti-PA8 and anti-TSKB20 IgG production, expansion of Ag-specific IFN-γ, TNF-α, and Granzyme B-producing CD8^+^ T cells, as well as *in vivo* Ag-specific cytotoxic responses. Moreover, the *fd*-TSKB20 vaccine was able to protect against mortality induced by a high-dose inoculum of the parasite. Although vaccination with r*fd* phages successfully reduced both parasitemia and parasite load in the myocardium of WT B6 mice, *Tlr9^−/−^* animals were not protected against infection. Thus, our data extend previous studies, demonstrating that r*fd* phages induce Ag-specific IgG and CD8^+^ T cell-mediated responses and confer protection against an important human parasite infection, through a TLR9-dependent mechanism.

## Introduction

Filamentous *fd* bacteriophages are non-lytic viruses that infect and replicate only in host bacteria and, therefore, have been considered safe for the vertebrate host so far. Both the minor coat protein involved in host cell recognition, pIII, and the major coat protein pVIII of *fd* phages can be fused to antigenic peptides (1). Previous studies have shown that antigen peptides expressed on the phage capsid can be displayed through MHC class I and class II pathways on antigen-presenting cells (APCs), resulting in the capacity of inducing immune responses mediated by specific antibodies and by helper and cytotoxic T lymphocytes (2, 3). Importantly, a single-chain antibody fragment (scFv), able to target dendritic cells (DCs) through the endocytic receptor DEC-205 (also known as CD205), can be introduced at the N-terminus of the pIII protein, further improving phage-uptake by DCs and their maturation. These *fd*sc-αDEC particles were shown to induced a strong and sustained CD8^+^ T cell-mediated anti-tumor response (1). Moreover, *fd*sc-αDEC virions were shown to activate the TLR9 pathway, which induces the maturation of DCs (4, 5).

The intracellular protozoan *Trypanosoma cruzi* is the etiologic agent of Chagas’ disease (American trypanosomiasis), which is the leading cause of myocardial disease and endemic in Latin America. Eight million people are estimated to be infected worldwide, of which 300,000 in the US, where enzootic cycles of *T. cruzi* have been recently established (6, 7). Since no mandatory screening exists for blood and tissue donors in non-endemic countries, it is expected that an altered epidemiology of Chagas disease will evolve considerably in a near future. No human vaccine is available to date and the approved drugs, nitrofuran and nitroimidazole, have limited efficacy in the chronic stage and important adverse side effects (8). Experimental *T. cruzi* infection in murine models has provided the means for the identification of protective immune responses, which need to be fully elucidated in order to allow the development of appropriated and safe human vaccines [reviewed in Ref. (9)]. Eliminating the parasite at the acute phase prevents parasite survival and may avoid chronic phase immunopathology. Therefore, prophylactic vaccination, by reducing or completely eliminating the parasite burden, represents a desirable method to restrict the development of the chronic symptoms of the disease. *T. cruzi* antigens recognized by immune sera from infected humans or animals served as the basis for studies employing recombinant proteins. These recombinant proteins included members of the large trans-sialidase (Ts) surface protein family, which are expressed mainly in the infective trypomastigote and amastigote forms of the parasite. Proteins belonged to the family of cysteine-proteases (cruzipain) and other antigens, such as the flagellar calcium-binding protein, paraflagellar rod protein-2, LYT-1 antigen, ribosomal protein L7a-like protein, and KMP11, among others, have also been used in different formulations and delivery systems, including recombinant proteins mixed with distinct adjuvants and platforms using DNA delivery or recombinant viruses [reviewed in Ref. (10)].

Here, we investigated the capacity of recombinant filamentous fd bacteriophages (r*fd*) phages to act as a delivery system for vaccination against the experimental infection with *T. cruzi* in mice, in the absence of any added adjuvant. For this, we genetically modified *fd* phages, by introducing the OVA_257–264_ peptide or the *T. cruzi* immunodominant peptides TSKB20 and PA8, derived from *trans*-sialidase (Ts) and amastigote surface protein-2 (ASP-2), respectively (11, 12), as an N-terminal fusion with the pVIII coat protein. Our results demonstrated that vaccination with r*fd* phages induces specific IgG and a strong CD8^+^ T cell-mediated response, enhancing the percentages of Ag-specific IFN-γ and TNF-secreting CD8^+^ T cells in the spleen, as well as the level of specific cytotoxicity *in vivo*. Accordingly, vaccinated mice displayed significant lower levels of parasitemia and parasite load in the myocardium and, moreover, exhibited an increased survival rate. Furthermore, we also demonstrated here that r*fd* phages devoid of the scFv anti-DEC-205 on pIII also require the expression of TLR9 by the host in order to confer protection against infection. Therefore, the present work extends previous studies on the immunogenicity mechanisms mediated by *fd* phages, reinforcing the potential use of these particles as a valuable delivery system for immunization without the need of exogenous adjuvant administration and shows, for the first time, its promising use in vaccination against intracellular parasites.

## Materials and Methods

### Construction of *fd-*PA8 and *fd-*TSKB20 Filamentous Bacteriophages

Oligonucleotide sequences encoding PA8 peptide (VNHRFTLV) from ASP-2 or TSKB20 peptide (ANYKFTLV) derived from *trans-*sialidase, flanked by the sequences for the +2 to +3 and +4 to +10 residues of the pVIII protein and by the 5′-protruding ends of SacII-StyI restriction sites were designed and purchased from Eurofins Genomics, Germany (PA8-up: 5′-GGAGGGTgt taaccaccgtttcaccctggttGACGATCCCGC-3′; PA8-dw: 5′-CTTGG CGGGATCGTCaaccagggtgaaacggtggttaacACCCTCCGC-3′; TSKb20-up: 5′-GGAGGGTgcgaactataaattcaccctggtgGAC GATCCCGC-3′; TSKb20-dw: 5′- CTTGGCGGGATCGTCcacc agggtgaatttatagttcgcACCCTCCGC-3′). Each pair of sequence was annealed and ligated into bacteriophage *fd*AMPLAY388-HA DNA previously digested with SacII and StyI restriction enzymes (1). The DNA were transformed into *Escherichia coli* TG1 recO cells, and their identities were confirmed by DNA sequencing.

### Purification of Bacteriophages Particles

Wild-type and hybrid *fd*-PA8, *fd*-TSKB20, and *fd*-OVA (expressing the recombinant OVA_257–264_-pVIII proteins) and *fd*-PA8αDEC (expressing also the recombinant scFv anti-DEC-205-pIII proteins) filamentous bacteriophages were purified from the supernatant of *Escherichia coli* TG1recO cells (1). Briefly, bacterial cells were grown in TY2X medium and the expression of the recombinant proteins was induced adding 0.1 mM isopropyl-beta–d-thiogalactopyranoside (Sigma-Aldrich) to the cultures. The bacteriophage virions were precipitated from *E. coli* supernatant using Polyethylene glycol 6000 (Sigma-Aldrich), purificated using cesium chloride (Sigma-Aldrich) gradient, and dialyzed against phosphate buffered saline (PBS) 1×. Elimination of LPS from phage particles was performed using Triton X-114 (Sigma-Aldrich) as previously described (13). Residual LPS contamination was assayed using the Limulus Amebocyte Lysate assay (Limulus Amebocyte Lysate QCL-1000 chromogenic modification, Lonza), according to the manufacturer’s instructions. The number of copies of pVIII displaying the exogenous peptides was estimated by N-terminal sequence analysis of the purified virions and resulted in 15–20% for *fd-*OVA and 40% for *fd-*PA8 and *fd-*TSKB20. The expression of the scFv anti-DEC-205 in the pIII protein of the purified virions was assessed by western blot analysis using a mouse anti-HA tag mAb (Roche-Boehringer, Basel, Switzerland).

### Mice and Ethics Statement

All animal experiments were conducted in accordance with guidelines of the Animal Care and Use Committee of the Federal University of Rio de Janeiro (Comitê de Ética do Centro de Ciências da Saúde CEUA-CCS/UFRJ). *Tlr9^−/−^* mice were generated by and obtained from Dr. S. Akira (Osaka University, Japan). Procedures and animal protocols were approved by CEUA-CCS/UFRJ license no.: IMPPG022.

### Parasite and Experimental Infection

Mice used for experiments were sex- and aged-matched, and housed with a 12-h light–dark cycle. Bloodstream trypomastigotes of the Y-strain of *T. cruzi* were obtained from Swiss mice infected 7 days earlier. The concentration of parasites was estimated and at least four mice per group were inoculated intraperitoneally (i.p.) with 2 × 10^3^ trypomastigotes (in 0.2 ml PBS), unless otherwise stated. The Y-OVA strain was obtained as follows: the *Ova* gene fragment was digested using XbaI and XhoI and then inserted into the pROCK-HYGRO vector, previously digested with the same restriction enzymes, to produce pROCK-Ova (14). *T. cruzi* Y-strain epimastigote forms were grown in liver infusion tryptose medium (pH 7.3) supplemented with 10% fetal bovine serum, streptomycin sulfate (0.2 g/l), and penicillin (200,000 units/l) at 28°C (all from Gibco Thermo Scientific). The parasite transfection was performed using electroporation following a previously described protocol (14). The transfected parasites were cultured for 6 weeks in the presence of hygromycin (200 µg/ml) (Sigma-Aldrich) for selection of parasites containing stably incorporated pROCK-Ova. For infection with Y-OVA strain, trypomastigotes were obtained from LLC-MK2-infected cultures and 2 × 10^6^ trypomastigotes (in 0.2 ml PBS) were injected i.p./mouse. Parasitemia was monitored by counting the number of bloodstream trypomastigotes in 5 µl of fresh blood collected from the tail vein. Mouse survival was followed daily. For tissue parasite load quantification, hearts of infected mice were excised after perfusion, minced and the cardiac tissue immediately homogenized in 1.0 ml of 4.0 M Guanidine thiocyanate (Sigma-Aldrich) containing 8.0 µl/ml of β-mercaptoethanol (Sigma-Aldrich) and processed for DNA extraction. Generation of PCR standards and detection of parasite tissue load by real-time PCR was carried out as described (15); briefly, primers amplify a repeated satellite sequence of *T. cruzi* DNA of 195 base-pairs: TCZ-Fwd: (5′-GCTCTTGCCCACAAGGGTGC-3′) and TCZ-Rev: (5′-CCAAGCAGCGGATAGTTCAGG-3′). Reactions with TNF-α-Fwd: (5′-CCTGGAGGAGAAGAGGAAAGAGA-3′) and TNF-α-Rev: (5′-TTGAGGACCTCTGTGTATTTGTCAA-3′) primers for *Mus musculus* TNF-α gene were used as loading controls. PCR amplifications were analyzed using primers in combination with SYBR Green^®^ on a StepOne Real-Time PCR System (Applied Biosystems, Life Technologies).

### Vaccination

Mice were injected i.p. with 100 µg of *fd* phages in 200 µl of PBS at day −17 and −7 and infected with *T. cruzi* at day 0, as illustrated on Figure S1A in Supplementary Material.

### Intracellular Cytokine Staining

Splenocytes isolated from infected mice were cultured in the presence of PA8 (VNHRFTLV) peptide at 3.0 µM, or left unstimulated, for 10 h at 37°C in the presence of 5.0 µM monensin (Sigma-Aldrich). Cells were surface stained with anti-CD8-PerCP, CD3-FITC, and H-2K^b^-PA8-biotinylated pentamers (Proimmune), followed by 20 min staining with SAv-BV605 and fixed for 10 min with a solution containing PBS, 4% paraformaldehyde at RT. Then, cells were permeabilized for 15 min with PBS, 0.1% bovine serum albumine, 0.1% saponin (Sigma-Aldrich). Intracellular cytokine staining was performed with anti-IFN-γ-PE-Cy7, anti-granzyme B (GzB)-APC, and anti-TNF-PE (all mAbs and SAv from Biolegend). At least 10,000 gated CD8^+^ lymphocyte events were acquired. Analytical flow cytometry was conducted with a FACSCantoII and the data were processed with FACSDiva™ software (BD Biosciences).

### *In Vivo* Cytotoxicity Assay

For the *in vivo* cytotoxicity assays, splenocytes of naive B6 mice were divided into three populations, each loaded with 2.5 µM of either H-2K^b^ -restricted OVA_257–264_ (SIINFEKL), or PA8 (VNHRFTLV), or TSKB20 (ANYKFTLV) peptides, or left untreated for 40 min at 37°C. During the last 10 min of incubation, each cell population was labeled with a different concentration of the fluorogenic dye CFSE (Molecular Probes Thermo Scientific) at final concentrations of either 8.6 µM (CFSE^high^), 2.45 µM (CFSE^int^), or 0.7 µM (CFSE^low^). Subsequently, each cell population was extensively washed and mixed with equal numbers of the other two cell populations, before being injected i.v. 15–20 × 10^6^ total cells per mouse. In each experiment, the same B6 (WT) CFSE-loaded target populations were injected in B6 and *Tlr9^−^*^/^*^−^* mice. Recipient animals were mice that had been vaccinated and infected (or not) with *T. cruzi* and naive controls. Spleen cells of recipient mice were collected 20 h after transfer, fixed with 1.0% paraformaldehyde and analyzed by cytometry, using a FACSCalibur Cytometer (BD Biosciences). Cells were gated on dot plot FSC × CFSE; percentages of CFSE^low^, CFSE^int^, and CFSE^high^ cells were obtained using CellQuest software (BD Biosciences). In all experiments, we refer to M1 as the control cell population without any exogenous peptide-loaded, and M2 and M3 are the cell populations loaded with different exogenous peptides. For calculating the percentage of specific lysis of each peptide-loaded population, M2 (or M3) was gated together M1, giving 100% of the events. Then, the following formula was applied: [1 − (M2_peptide-loaded_ or M3_peptide-loaded_ in Experimental group/M1_without Ag peptide_ in Experimental group)/(M2_peptide-loaded_ or M3_peptide-loaded_ in CTR group/M1_without Ag peptide_ in CTR group)] × 100%; Experimental group are either vaccinated mice, infected mice, or both vaccinated and infected mice; CTR group are naive mice.

### Anti-Mouse IgG ELISA

Sera from naive B6 mice, *fd-*WT (ctr) and *fd-*PA8 or *fd*-TSKB20 vaccinated and infected mice were diluted 1:5,000 and adsorbed overnight (ON) at 4°C, on treated microplates (Corning-Costar) previously coated with *fd*-WT bacteriophages immobilized by polyclonal rabbit anti-fd phage IgG (Sigma-Aldrich). After absorption against *fd*-WT antigens, the sera were harvested and tested for reactivity against *fd*-WT and *fd-*PA8 or *fd*-TSKB20 antigens. Briefly, the absorbed sera were incubated at different dilutions overnight at 4°C, on microplates previously coated with *fd*-WT or *fd*-PA8 or *fd*-TSKB20 particles; secondary goat anti-mouse IgG antisera (1:2,500), labeled with peroxidase (HRP) (SouthernBiotech), were added for 4 h at room temperature and the reaction revealed with TMB (Thermo Scientific).

### Bone Marrow-Derived Dendritic Cell (BMDC) Culture

Bone marrow-derived dendritic cells were obtained as previously described (16). Briefly, BM cells were cultured in tissue-culture-treated flasks at 1 × 10^6^/ml in complete RPMI 1640 medium, supplemented with 10% heat-inactivated fetal calf serum (all from GIBGO, Thermo Scientific), and 20 ng/ml rmGM-CSF (R&D Systems), at 37°C, 5% CO_2_ humid atmosphere. On day 2 of culture, 3 ml of the medium was removed and fresh warmed medium supplemented with GM-CSF (2×, 40 ng/ml) added. At day 3, medium was entirely replaced by fresh warmed medium with GM-CSF (20 ng/ml). On day 6, loosely adherent cells were harvested by gentle washing with PBS and cultured for 20 h in 96-well microplates in the presence of the indicated reagents (LPS, CpG, and *fd*-WT phages at 100 ng/ml, 1.0 µg/ml, and 250 µg/ml, respectively) and subsequently stained for flow cytometry analysis. LPS (*E. coli* 055:B5 strain) and CpG (ODN D-SL03) were from InvivoGen (San Diego, CA, USA).

### Confocal Microscopy

LLC-MK2 cells were infected with cell-culture derived Y-strain trypomastigotes at 10:1 ratio overnight at 37°C, 5% CO_2_. After 2 days, cells were fixed in 4% paraformaldehyde/PBS for 15 min and permeabilized with three washes with PBS-0,1% Triton X-100 (Bio-Rad). Sera of immunized and/or infected mice, as well as of naïve animals were added and incubated overnight at 4°C. After washing three times with PBS-0, 1% Triton X-100, donkey Cy3-labeled anti-mouse IgG was added for 2 h at RT. After washing, DAPI was added at 1 µg/ml at RT for 5 min. The coverslips were assembled with vectashield and fixed with enamel. Confocal microscopy was performed with a Zeiss Axio Observer.Z1 inverted microscope equipped with a CSU-X1A 5000 Yokogawa Spinning Disk confocal unit using a 100 × NA 1.4, oil-immersion plan-apochromatic objective. Images were captured with QImaging Rolera EM-C2 camera using Zen 2.3 system (Zeiss), and processed off line with Photoshop.

### Database Search

The PA8 peptide (VNHRFTLV) sequence was queried at 100% coverage in the NCBI Protein Reference Sequence Database using Basic Local Alignment Search Tool (BLAST)^1^ in order to identify proteins expressed by *T. cruzi* that could contain this epitope, other than the amastigote ASP-2 protein.

### Statistical Analysis

Statistical analyses were performed using GraphPad Prism version 4.00 for Windows (GraphPad Software, San Diego California USA^2^). Data were compared using a two-tailed Student’s *t* test and are expressed as mean ±SEM. Data were considered statistically significant if *p* values were <0.05. The Gehan–Breslow–Wilcoxon test was used to compare the mouse survival rate. The differences were considered significant when the *p* value was <0.05.

## Results

### *fd-*OVA_257–264_ Phage Protects B6 but Not *Tlr9^−/−^* Mice Against Infection With *T. cruzi* Y-OVA Strain

**Figure 1:**
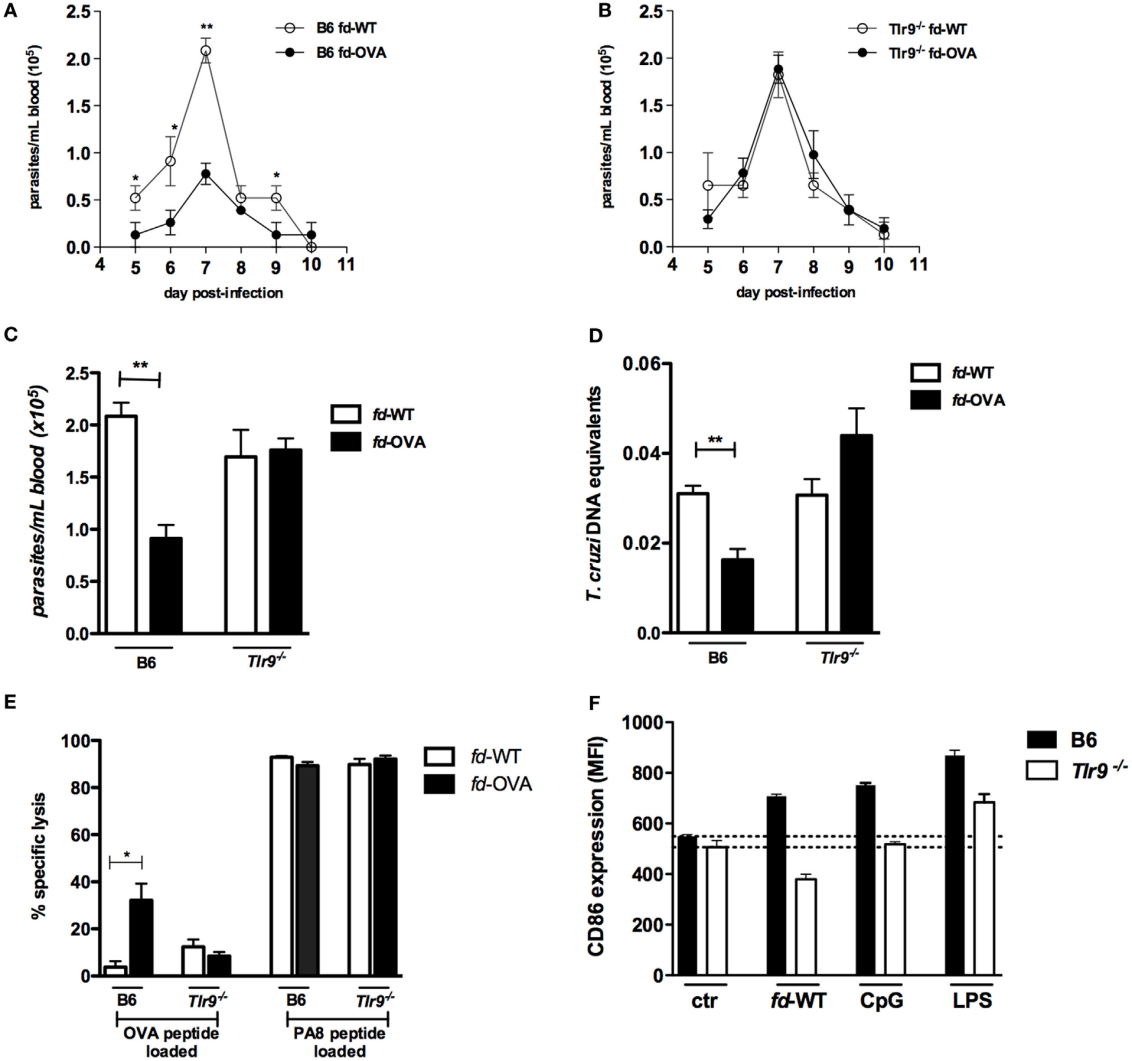
Immunization with *fd-*OVA phages protects against infection with *T. cruzi* Y-OVA strain in TLR9-dependent manner. Male mice were immunized (as in Figure S1 in Supplementary Material) and infected on day 0 with 2 × 10^6^ culture trypomastigotes of Y-OVA strain. Parasitemia curves of **(A)** B6 mice and **(B)** *Tlr9^−/−^* immunized with *fd*-OVA (black circles), or *fd*-WT phages as control (empty circles). Mean parasitemia values at day 7 pi. **(C)**. Parasite load in the myocardium at day 20 pi **(D)** and Ag-specific cytotoxicity *in vivo* against target cells loaded with OVA or ASP-2-derived (PA8) peptides, B6 and *Tlr9^−/−^* mice were immunized with *fd*-OVA (black bars) or *fd*-WT (white bars) (representative contour plots are shown in Figure S1B in Supplementary Material) **(E)**. CD86 expression (MFI) on bone marrow-derived dendritic cell (BMDC) after 20 h treatment *in vitro* with *fd*-WT (250 µg/ml), CpG (1.0 µg/ml), LPS (100 ng/ml) or left untreated (ctr). B6 (black bars) and *Tlr9^−/−^* (white bars) BMDCs **(F)**. Data represent mean values of individually analyzed mice (*n* = 5 per group) from control or immunized mice **(A–E)** or triplicate cultures **(F)**. Error bars = SEM, *p ≤ 0.05; **p ≤ 0.01 (two-tailed Student’s *t*-test).

To investigate whether vaccination with phage particles would protect against infection with *T. cruzi*, we first immunized B6 (WT) mice with the model antigen ovalbumin (OVA_257–264_) SIINFEKL peptide, a known H-2K^b^-restricted epitope, as an N-terminal fusion with the pVIII phage protein (*fd-*OVA). After vaccination, mice were infected with the *T. cruzi* Y-OVA transgenic strain, following the immunization and challenge scheme shown in Figure S1A in Supplementary Material; injection of *fd-*WT phages, which do not express any *T. cruzi* antigen, was employed as control treatment. As shown in Figure 1A, the level of blood parasitemia is significantly lower in B6 mice immunized with *fd*-OVA, when compared to B6 mice immunized with *fd-*WT phage. On the other hand, immunization was not able to reduce parasitemia levels in *Tlr9^−/−^* mice (Figures 1B,C). The same was true when parasite loads in the myocardium were measured at day 20 post-infection (pi) by qPCR (Figure 1D). We then investigated the levels of specific *in vivo* cytotoxicity against target cells loaded with the OVA_257–264_ SIINFEKL peptide at day 20 pi in mice vaccinated with *fd*-OVA or *fd*-WT phages. As shown in Figure 1E, while *fd*-OVA-vaccinated B6 (WT) mice displayed around 30% of specific lysis against SIINFEKL-loaded targets, no significant increase in Ag-specific cytotoxicity was found in immunized *Tlr9^−/−^* mice (representative dot blots are shown on Figure S1B in Supplementary Material). However, no difference in the levels of specific cytotoxicity against the endogenous immunodominant *T. cruzi*-derived PA8 (VNHRFTLV) peptide was observed between WT and *Tlr9^−/−^* mice, immunized with *fd*-OVA or not (Figure 1E), in accordance to our previous data showing that the cytotoxic response is preserved in infected *Tlr9^−/−^* mice (17). Therefore, these results demonstrate that while effective in protecting WT B6 mice, the vaccination with *fd*-OVA did not confer any protection to *Tlr9^−/−^* mice against infection with *T. cruzi* Y-OVA strain.

### *fd* Phage Particles Induce the Maturation of DCs Through a TLR9-Dependent Pathway

We next tested the *fd* virion property of inducing BMDC maturation *in vitro*. For this, WT (B6) and *Tlr9^−/−^* BMDC were cultured for 20 h in the presence of *fd*-WT particles or of the TLR9 and TLR4 ligands CpG and LPS, respectively. As shown in Figure 1F, *fd* phages were able to induce the upregulation of the CD86 costimulatory molecule in WT, but not in *Tlr9^−/−^* BMDCs. Note that, here, a 2.5 higher dose of *fd* phage particles was employed compared to the dose used in a previous study (1). Together, these data demonstrated that *fd-*OVA phages devoid of sc-αDEC also depend on the TLR9 pathway in order to induce the maturation of DCs and, consequently, a specific cytotoxic T cell (CTL) response, as previously shown for *fd*-OVA-αDEC virions (4).

### Vaccination With r*fd* Phages Displaying the *T. cruzi* Immunodominant PA8 Peptide Protect B6 Mice Against Infection

**Figure 2:**
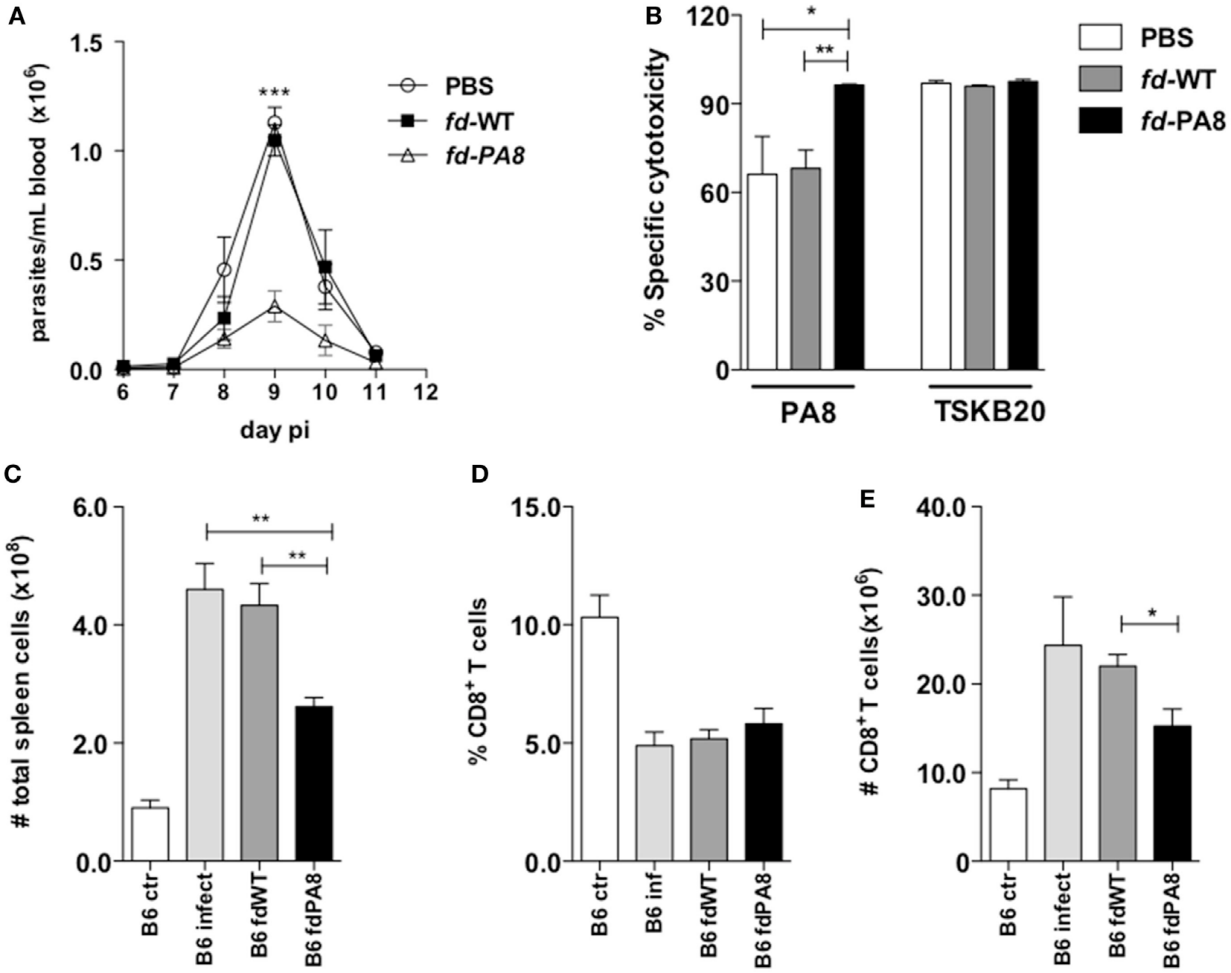
Immunization with *fd-*PA8 phages protects B6 mice against infection with *T. cruzi* Y-strain and increments Ag-specific cytotoxicity. Male mice were immunized (as in Figure S1 in Supplementary Material) and infected on day 0 with 2 × 10^3^ blood trypomastigotes of Y-strain. Parasitemia curves **(A)** and specific cytotoxicity against target cells loaded with PA8 or TSKB20 peptides (representative contour plots are shown in Figure S2 in Supplementary Material) **(B)**, of B6 mice immunized with *fd*-PA8 (triangles and black bars), or *fd*-WT phages (black squares and gray bars), or only infected [phosphate buffered saline (PBS)] (empty circles and white bars) as controls. Total spleen cell numbers **(C)**, CD8^+^ T cell percentages **(D)**, and absolute CD8^+^ T cell numbers **(E)**. Data represent mean values of individually analyzed mice (*n* = 5 per group) from control naïve (white bars), infected-only mice (light gray bars), *fd*-WT (dark gray bars) or *fd*-PA8 immunized mice (black bars). Error bars = SEM, *p ≤ 0.05; **p ≤ 0.01; ***p ≤ 0.001 (two-tailed Student’s *t*-test).

**Figure 3:**
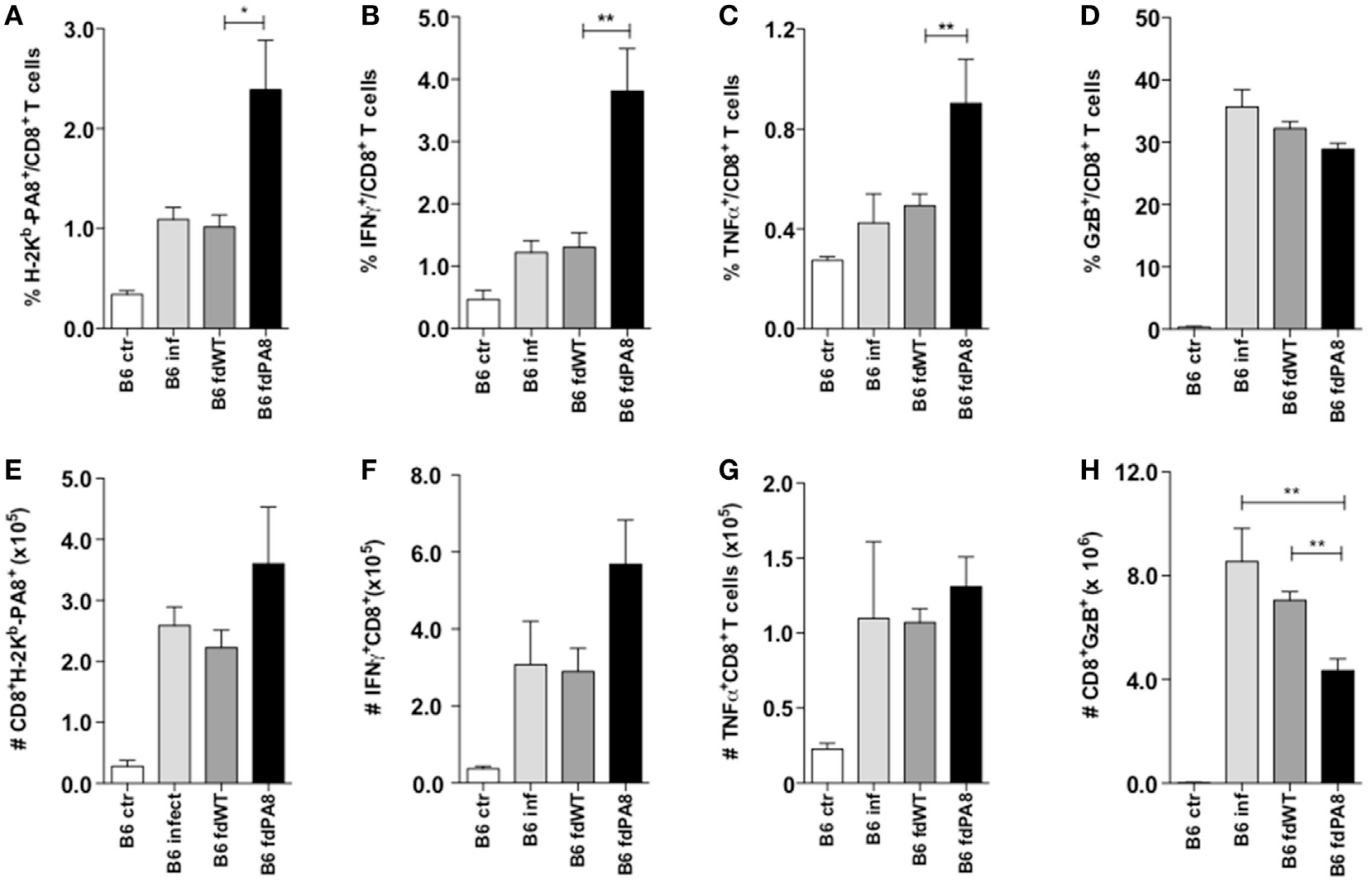
Immunization with *fd-*PA8 phages increases the levels of Ag-specific and cytokine-secreting CD8^+^ T cells in the spleen of B6 mice. Male mice were immunized (as in Figure S1 in Supplementary Material) and infected on day 0 with 2 × 10^3^ blood trypomastigotes of the Y-strain. On Day 13 pi, splenocytes from control naïve (white bars), infected-only (light gray bars), *fd*-WT (dark gray bars), or *fd*-PA8 immunized mice (black bars) were stained following a 10-h *in vitro* incubation with PA8 peptide, as described in Section Materials and Methods. Mean percentages **(A–D)** and absolute numbers **(E–H)** of H-2K^b^-PA8^+^ **(A,E)**, IFN-γ^+^ **(B,F)** TNF^+^ **(C,G)**, and GzB^+^ **(D,H)** CD8^+^ T cells of individually analyzed mice (*n* = 5) are shown. Error bars = SEM, *p ≤ 0.05; **p ≤ 0.01 (two-tailed Student’s *t*-test).

The *T. cruzi* ASP-2-derived PA8 peptide was described as an immunodominant peptide, which induces high levels of CD8^+^ CTLs (11). We then constructed r*fd* phages expressing the PA8 peptide and use it to vaccinate B6 mice, following the same previous protocol (Figure S1 in Supplementary Material). As shown in Figure 2A, the parasitemia was significantly diminished in mice previously vaccinated with *fd*-PA8, but not with *fd*-WT phages, when compared to parasitemia in non-vaccinated mice (PBS group). The *in vivo* cytotoxicity assay revealed an increased lysis rate against target cells loaded with the PA8 peptide in *fd*-PA8-vaccinated mice at day 13 pi, while no differences in the level of cytotoxicity against control (TSKB20) immunodominant peptide-loaded target cells was found between the groups (Figure 2B; Figure S2 in Supplementary Material). In accordance with the lower parasite load, the spleen of *fd*-PA8-vaccinated mice display lower numbers of CD8^+^ T cells, as well as of total splenocytes, although the percentage of total CD8^+^ T cells is equal in all the infected groups (Figures 2C–E). We also analyzed the expansion of PA8-specific CD8^+^ T cells by staining with K^b^-PA8 pentamers and found that their frequency was increased by *fd*-PA8 vaccination (Figures 3A,E; contour plots shown in Figure S3 in Supplementary Material). Then, we investigated the induction of CTL and cytokine-secreting CD8^+^ T cells in the spleens of *fd*-PA8 or *fd*-WT vaccinated and infected mice, as well as in only infected (B6 infect) and non-infected (B6 ctr) mice. For this, the percentages (Figures 3B–D) and absolute numbers (Figures 3F–H) of CD8^+^ T cells expressing IFN-γ, TNF, or GzB were assessed by *ex vivo* restimulation of splenocytes with the PA8 peptide, followed by intracellular staining. As shown in Figures 3B,C, vaccination with *fd*-PA8 increased the percentages of cytokine-secreting CD8^+^ T cells, while the percentages of IFN-γ^+^ CD8^+^ and TNF^+^ CD8^+^ T cells is not different between *fd*-WT-treated and non-treated infected mice. On the other hand, no difference was found on the percentages of GzB^+^ cells between the different groups of infected mice (Figure 3D). As the absolute number of CD8^+^ T cells is lower in *fd*-PA8-vaccinated mice (Figure 2E), significant lower numbers of GzB^+^ CD8^+^ T cells are found in this group (Figure 3H).

**Figure 4:**
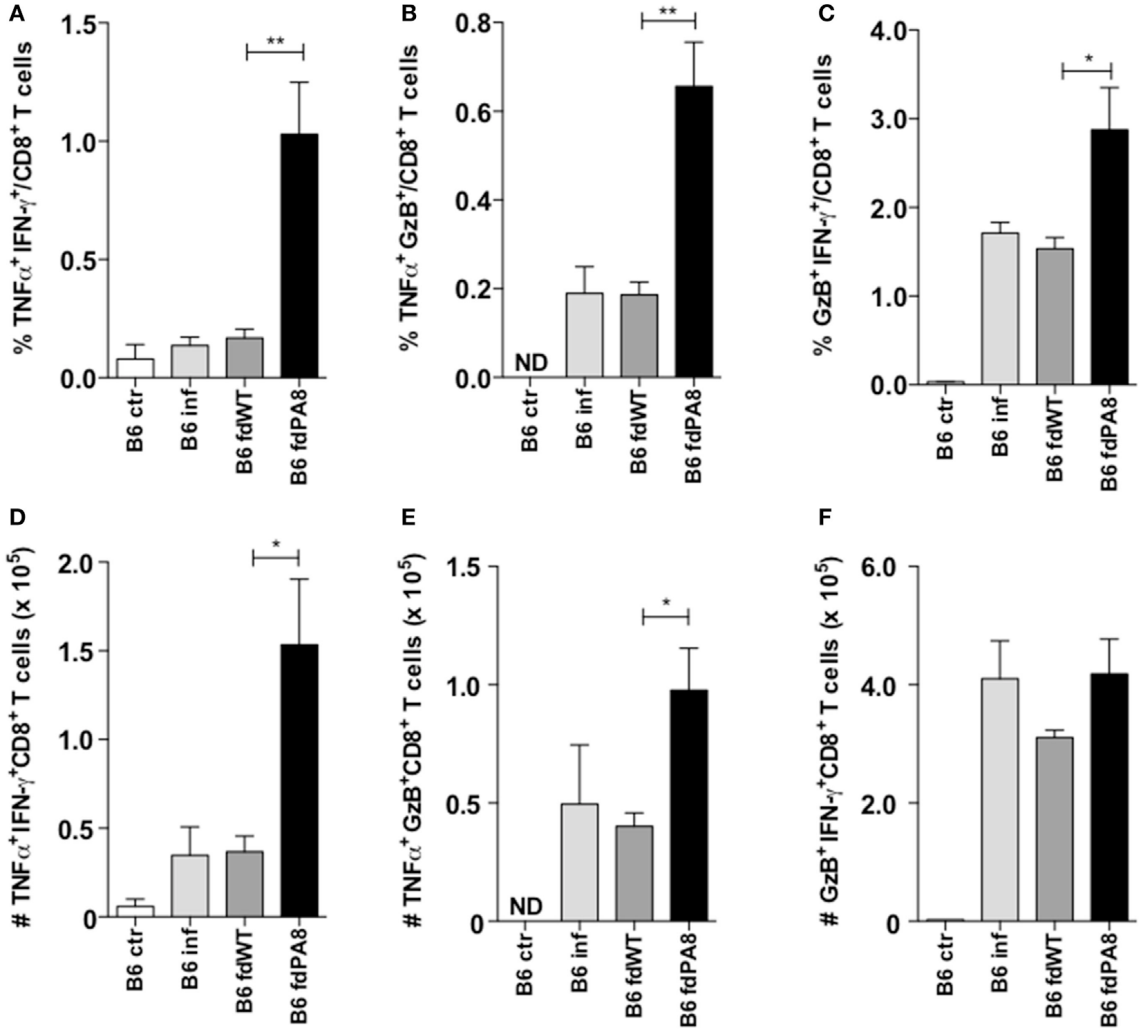
Immunization with *fd-*PA8 phages increases the levels of polyfunctional CD8^+^ T cells in the spleen of B6 mice. Male mice were immunized (as in Figure S1 in Supplementary Material) and infected on day 0 with 2 × 10^3^ blood trypomastigotes of the Y-strain. On Day 13 pi, splenocytes from control naïve (white bars), infected-only (light gray bars), *fd*-WT (dark gray bars), or *fd*-PA8 immunized mice (black bars) were stained following a 10-h *in vitro* incubation with PA8 peptide, as described in the Section Materials and Methods. Mean percentages **(A–C)** and absolute numbers **(D–F)** of TNF^+^IFN-γ^+^ **(A,D)**, TNF^+^GzB^+^ **(B,E)**, and IFN-γ^+^GzB^+^ **(C,F)** CD8^+^ T cells of individually analyzed mice (*n* = 5) are shown. Error bars = SEM, *p ≤ 0.05; **p ≤ 0.01 (two-tailed Student’s *t*-test).

Since it has been described that infected mice previously vaccinated with a recombinant adenovirus expressing ASP-2 display higher levels of polyfunctional CD8^+^ T cells, compared to non-vaccinated infected mice (18), we also analyzed here the percentages and absolute numbers of CD8^+^ T cells producing simultaneously TNF and IFN-γ, or TNF and GzB, or GzB and IFN-γ in the four experimental groups (naïve controls, infected-only, *fd*-WT + infection, and *fd*-PA8 + infection). As shown in Figure 4, all these subsets of double-positive CD8^+^ T cells are increased in the spleens of infected mice previously immunized with *fd*-PA8 phages. Moreover, the frequency of triple positive (IFN-γ^+^TNF^+^GzB^+^) CD8^+^ T cells is also increased in the *fd*-PA8-vaccinated group, as shown in Figures S4A–C in Supplementary Material. Therefore, our results suggest that, contrary to what happens in non-vaccinated infected mice, vaccination with r*fd* phages causes the expansion of polyfunctional CD8^+^ T cells, which correlates to protection against infection with *T. cruzi*.

### Previous Immunization With *fd*-PA8 Induces Higher Levels of Peptide-Specific IgG Antibodies in Infected Mice

**Figure 5:**
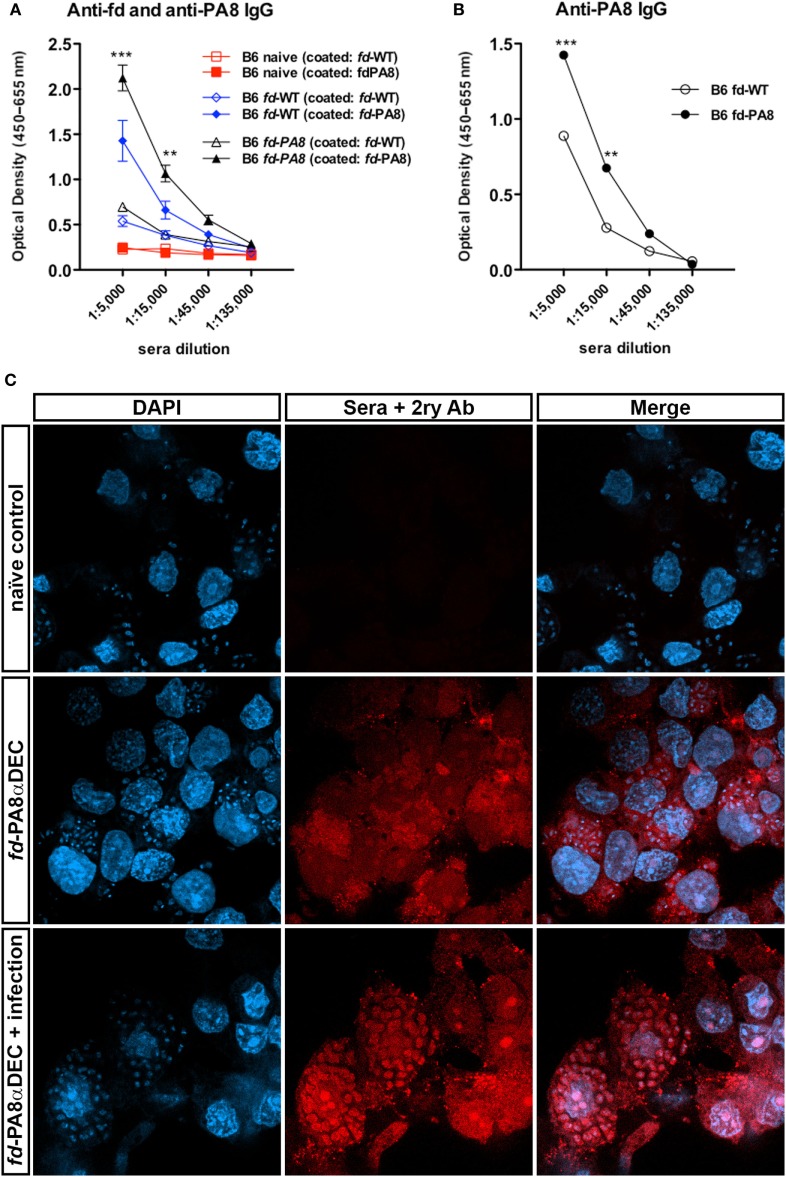
Immunization with *fd-*PA8 phages increases the levels of PA8-specific IgG in the serum B6 mice. Male mice were immunized (as in Figure S1 in Supplementary Material) and infected on day 0 with 2 × 10^3^ blood trypomastigotes of the Y-strain. On Day 13 pi, sera were collected from control naïve (red symbols and lines), *fd*-WT (blue symbols and lines), and *fd*-PA8-immunized mice (black symbols and lines), adsorbed against immobilized *fd*-WT phages and then tested on an ELISA assay against *fd*-WT and *fd*-PA8 phages, as described in the Section “Material and Methods.” Mean values (*n* = 3), error bars = SEM **(A)**. OD curves obtained against *fd*-WT phage was subtracted from OD curves obtained against *fd*-PA8 phages; **p ≤ 0.01; ***p ≤ 0.001 (two-tailed Student’s *t*-test) **(B)**. Confocal microscopy of LLC-MK2 cells infected with Y-strain at day 2 pi **(C)**. Cells were stained with serum from control naïve or *fd*-PA8αDEC-immunized-only or *fd*-PA8αDEC-immunized and infected mice, followed by secondary anti-mouse IgG Cy3-labeled Ab and DAPI, as detailed in the Section “Materials and Methods.”

Since vaccination using *fd* phages as delivery system can also induce the production of Ag-specific antibodies (2, 19), we also quantified here the PA8-specific IgG in the sera of vaccinated and *T. cruzi*-challenged B6 mice. For this, sera of naïve controls, *fd*-WT + infection-, and *fd*-PA8 + infection-groups were collected at day 13 pi and first absorbed against immobilized *fd*-WT phages, in order to (at least partially) deplete anti-phage Igs. Then, each *fd*-absorbed serum was further tested in an ELISA assay against either immobilized *fd*-PA8 or *fd*-WT particles (Figure 5A). The difference in the obtained optical densities testing absorbed *fd-*PA8 sera against *fd*-PA8- and *fd*-WT-coated phages represents the titer of specific anti-PA8 IgG. We found that PA8-specific IgG Abs are also present in mice immunized with *fd*-WT and challenged with infection, although in these mice PA8-specific IgG Abs are at significantly lower levels to the ones found in mice vaccinated with *fd*-PA8 and infected, as quantified by ELISA assays (Figures 5A,B). In order to confirm that PA8 peptide-specific IgG antibodies induced by vaccination with *fd*-PA8 phages are capable of recognizing ASP-2 protein on the amastigote forms, sera from *fd*-PA8αDEC immunized-only (non-infected) mice were also employed in the immunofluorescence assays against the *in vitro* infected LLC-MK2 cell line (Figure 5C). These data indicate that the immune sera from vaccinated mice contain peptide-specific IgG Abs able to recognize the amastigote antigen. Since the ASP-2 molecule is a member of the *T. cruzi trans*-sialidase (Ts) superfamily, we hypothesized that other proteins of this family could also contain the same peptide sequence. We then searched the non-redundant NCBI Protein Reference Sequence Database using BLAST. The obtained result is displayed in Table S1 in Supplementary Material: the PA8 sequence (VNHRFTLV) was found at 100% coverage and 100% identity in six different putative Ts protein sequences (20). Search of the UniProtKnowledgebase using Peptide Search,^3^ gave the same result (not shown). Hence, this epitope is also present in other proteins potentially expressed at the surface of trypomastigotes (the parasite infecting forms) and, as such, might be recognized by neutralizing and/or opsonizing antibodies (21, 22).

### Vaccination With r*fd* Phages Displaying the TSKB20 Peptide Reduces Mortality and Increases Specific Cytotoxicity Long Term After Infection

**Figure 6:**
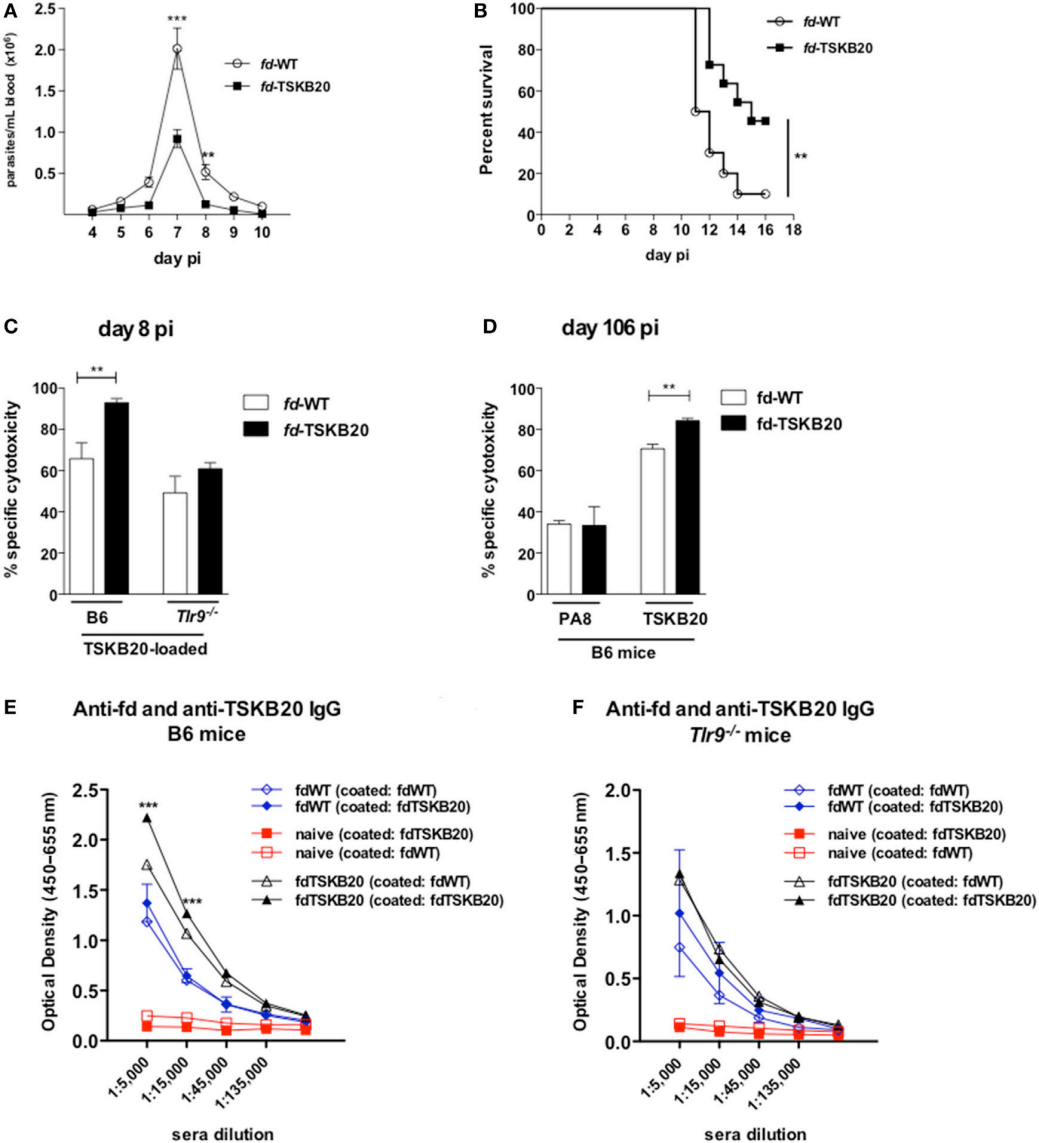
Immunization with *fd-*TSKB20 phages protects against infection with *T. cruzi* Y-strain and increases IgG and Ag-specific cytotoxicity in TLR9-dependent manner. Male mice were immunized (as in Figure S1 in Supplementary Material) and infected on day 0 with 2 × 10^5^ blood trypomastigotes of Y-strain. Parasitemia curves (*n* = 6 in each group) **(A)** and survival curves **(B)** of B6 mice immunized with *fd*-TSKB20 (black squares) or *fd*-WT phages (empty circles), as controls (*n* = 10 in each group) **p ≤ 0.01 (Gehan–Breslow–Wilcoxon Test). Specific cytotoxicity in B6 and *Tlr9^−/−^* mice at day 8 pi, against target cells loaded with TSKB20 peptide (*n* = 4) **(C)**. Specific cytotoxicity in B6 mice at day 106 pi against target cells loaded with PA8 or TSKB20 peptides. Bars represent mean values (*n* = 3) **(D)**. **(A,C,D)** Error bars = SEM; **p ≤ 0.01; ***p ≤ 0.001 (two-tailed Student’s *t*-test). On Day 8 pi, sera were collected from control naïve (red symbols and lines), *fd*-WT (blue symbols and lines), and *fd*-TSKB20 immunized (black symbols and lines) B6 mice **(E)** or *Tlr9^−/−^* mice **(F)**, adsorbed against immobilized *fd*-WT phages and then tested on an ELISA assay against *fd*-WT and *fd*-PA8 phages, as described in the Section “Materials and Methods.” Symbols represent mean values (*n* = 3), error bars = SEM; ***p ≤ 0.001 (two-tailed Student’s *t*-test).

We next constructed r*fd* phages displaying the previously described K^b^-restricted immunodominant TSKB20 peptide (ANYKFTLV) derived from the Ts of *T. cruzi* (12). In order to follow mortality, a higher inoculum of parasite (2 × 10^5^ blood trypomastigotes) was employed in the experiment illustrated in Figure 6. Apart from that, mice were immunized twice with *fd*-TSKB20 following the same vaccination/infection scheme showed in Figure S1 in Supplementary Material. As shown in Figure 6A, vaccination with *fd*-TSKB20 significantly decreased parasitemia levels in B6 mice. Moreover, while only 10% of B6 mice immunized with *fd*-WT survived infection, around 50% of mice vaccinated with *fd*-TSKB20 were protected (Figure 6B). Then, we tested the *in vivo* cytotoxicity against TSKB20-loaded target cells both in B6 and *Tlr9^−/−^* vaccinated mice at an early time point (day 8 pi) after the challenging infection. As shown in Figure 6C, vaccination increased the specific cytotoxic response in WT, but not in *Tlr9^−/−^*-vaccinated mice. It is known that the CTL response against certain immunodominant peptides last for hundred of days in B6 mice infected with the *T. cruzi* Y-strain (23). Accordingly to this, we could detect *in vivo* Ag-specific cytotoxicity against both PA8 and TSKB20 peptides at day 106 pi (Figure 6D). However, only the CTL response against TSKB20-pulsed target cells was significantly increased in mice vaccinated with *fd*-TSKB20 (Figure 6D). Finally, we have also investigated the early production of anti-TSKB20 IgG in vaccinated mice, at day 8 pi. As shown in Figures 6E,F, TSKB20-specific IgG could be detected in the sera of vaccinated B6 but not *Tlr9^−/−^* mice. Therefore, together these results demonstrate that vaccination with *fd*-TSKB20, as well as with *fd*-PA8, confers protection against infection with the *T. cruzi*, as it diminishes parasitemia and increases survival, being able to induce a more robust long-lasting CTL response, as well as early IgG production, against specific parasite epitopes. Moreover, both effector anti-parasite mechanisms, CTL and IgG responses, induced by r*fd* phages, were shown to depend on the activation of the *Tlr9*-mediated innate pathway.

## Discussion

Filamentous *fd* phages are viruses which structure is formed by a cylindrical flexible protein scaffold, approximately 7-nm wide and 890-nm long, containing a single-strand DNA genome rich in CpG motifs (24). It has been previously shown that recombinant *fd* phages, expressing a CD8 epitope and directed to CD205^+^ DCs (*fd*sc-αDEC), are potent inducers of Ag-specific CTL responses and are more effective than other immunization strategies for inhibiting the growth of the B16 tumor *in vivo* (1), reviewed in Ref. (24). In fact, DCs show an enhanced receptor-mediated binding and internalization of phage particles expressing the anti-DEC-205 scFv, when compared to *fd*-WT virions, using both *in vitro* and in *in vivo* assays (1). Moreover, it has been recently demonstrated that *fd*sc-αDEC virions are delivered to the late endosome/lysosome compartment and induce the activation of the TLR9 pathway in DCs, which, as a consequence, secrete different pro-inflammatory cytokines, including type I IFN, leading to DCs maturation (4). Although *fd* phages devoid of sc-αDEC are not as efficient as *fd*sc-αDEC in inducing the maturation of DCs, we have shown here, by employing phage doses that are 2- to 2.5-fold higher than the previously used (1, 4), that r*fd* phages devoid of sc-αDEC nevertheless induce the expression of the costimulatory molecule CD86 in WT BMDCs, but not in TLR9-deficient cells. Importantly, we have also shown here that vaccination with these r*fd* particles is capable to induce the protection of B6 mice against infection with the intracellular *T. cruzi* parasite, as it reduces parasitemia, parasite load in the myocardium and mortality in WT-infected mice. However, none of these infection parameters was reduced in vaccinated *Tlr9^−/−^* mice. This was shown using either *fd*-OVA followed by infection with the transfected Y-OVA strain of *T. cruzi*, as a proof of concept, or *fd* phages expressing the *T. cruzi trans*-sialidase-derived immunodominant epitope TSKB20 followed by infection with the *T. cruzi* Y-strain. It is not clear, at the moment, if TLR9-signaling is required only for the induction of type I IFN and costimulatory molecules in DCs or if it also increases the efficiency of the parasite-antigen cross-presentation. This is an interesting point and deserves further work in order to be elucidated.

Regarding the effector immune mechanisms induced by vaccination with r*fd* phages, which would be responsible for the protection against infection with *T. cruzi*, we have shown here that vaccination induces higher levels of Ag-specific and cytokine-secreting CD8^+^ T cells, as well as higher levels of Ag-specific cytotoxicity *in vivo*. The CTL response, as well as the secretion of IFN-γ by CD8^+^ T cells, has been known to play a fundamental role on the protection against infection with *T. cruzi* and with other intracellular pathogens; reviewed in Ref. (10). Of note, we have found that immunization with r*fd* phages increases the percentages and absolute numbers of polyfunctional CD8^+^ T cells in the spleen of vaccinated mice. This phenomenon was previously observed in other models of experimental vaccination, such as against *T. cruzi*, employing adenovirus as a vaccine vector (18) and against malaria, using prime-boost immunization with modified vaccinia virus Ankara and adenoviral vectors (25). Moreover, polyfunctional human CD8^+^ T cells are found at higher levels in HIV nonprogressors than in progressors and the presence of these multifunctional T cells negatively correlates with viral load in the latter group (26, 27). Nevertheless, until the present date, it has not been possible to clearly attribute a role to polyfunctional T cells as markers of protective immunity in other infections (28), and therefore, more studies are necessary to clarify this point. It is known that the parasite displays escape mechanisms that retard and somehow compromises the immune response toward it, which leads to the chronification of the infection. Thus, it is possible that vaccination is beneficial not only by inducing an immune response that precedes infection but also because it allows the establishment of a robust and qualitatively superior multifunctional CD8^+^ T cell-mediated response, which correlates to protection in some infection models.

On the other hand, we have also found here that immunization with *fd*-PA8 and *fd*-TSKB20 phages induces increased levels of serum IgG Abs directed against these ASP-2- and *trans*-sialidase (Ts)-derived parasite epitopes. The capacity of r*fd* phages in inducing a humoral response to vaccine Ags has been previously described (2, 19). Of note, both the ASP-2 and Ts proteins belong to an extended multigene family, which members are expressed at the cell surface at different stages of the protozoan life cycle and against which Abs might cross-react. More than 1,400 copies of the Ts gene were found in the *T. cruzi* genome (of which almost the half are pseudogenes), encoding full length and partial non-enzymatically active Ts molecules, the exact function of which is still not clear (20). In the absence of active Ts enzymatic function, trypomastigotes invade host cells poorly and are highly sensitive to host complement-mediated lysis (29). Moreover, several studies have suggested an immunomodulatory role for proteins of the Ts family (30). Hence, it is possible that anti-PA8 IgG would also bind to other Ts protein family members expressed on the surface of the infective trypomastigote stage (as indicated by the search with PA8 peptide query against the NCBI database), and consequently, would lead to parasite opsonization and neutralization. In fact, IgG2a lytic and opsonizing Abs have been described as important effector mechanisms against infection with *T. cruzi* (21, 22). Most probably, both humoral and cell-mediated responses contribute to the effective protection induced by r*fd* vaccination. This issue is currently under investigation by our group.

Finally, it is interesting to note that the induction of not only the CD8^+^ T cell-mediated cytotoxicity, but also the anti-TSKB20 IgG response induced by previous vaccination with *fd*-TSKB20 was defective in *Tlr9^−/−^* mice. This is probably the consequence of a poor innate activation of DCs in *Tlr9^−/−^* mice, leading to a defective activation of T-follicular helper cells, which in turn are required for helping the B cell response. It is tempting to speculate that *fd* phages might also directly interfere with the B-cell response through the B-cell intrinsic activation of the TLR9 pathway.

In summary, the results obtained in the present work further extend previous studies, demonstrating that r*fd* phages can be used as potent and innovative delivery systems, conferring both humoral and cellular-mediated protection against an important human parasite infection, through a TLR9-dependent mechanism.

## Ethics Statement

All animal experiments were conducted in accordance with guidelines of the Animal Care and Use Committee of the Federal University of Rio de Janeiro (Comitê de Ética do Centro de Ciências da Saúde CEUA-CCS/UFRJ). Procedures and animal protocols were approved by CEUA-CCS/UFRJ license no.: IMPPG022.

## Author Contributions

JG-N, AN, PB, and MB conceived and designed the experiments. JG-N, RS, FC, C-HB, TA, AD, PA, GM, and MS performed the experiments. JG-N, FC, MS, AN, and MB analyzed the data. AO, CM, HG, MS, AN, PB, and MB contributed parasites/reagents/mice/materials/analysis tools. MB wrote the paper.

## Conflict of Interest Statement

The authors declare that the research was conducted in the absence of any commercial or financial relationships that could be construed as a potential conflict of interest.

## Footnotes

^1^www.ncbi.nlm.nih.gov/BLAST/ (Accessed: January 10, 2018).

^2^www.graphpad.com (Accessed: January 10, 2018).

^3^http://www.uniprot.org/peptidesearch/ (Accessed: January 10, 2018).

## References

[1] Sartorius R Bettua C D’Apice L Caivano A Trovato M Russo D Vaccination with filamentous bacteriophages targeting DEC-205 induces DC maturation and potent anti-tumor T-cell responses in the absence of adjuvants. Eur J Immunol (2011) 41(9):2573–84. 10.1002/eji.201141526 21688262

[2] De Berardinis P D’Apice L Prisco A Ombra MN Barba P Del Pozzo G Recognition of HIV-derived B and T cell epitopes displayed on filamentous phages. Vaccine (1999) 17(11–12):1434–41. 10.1016/S0264-410X(98)00377-6 10195779

[3] De Berardinis P Sartorius R Caivano A Mascolo D Domingo GJ Del Pozzo G Use of fusion proteins and procaryotic display systems for delivery of HIV-1 antigens: development of novel vaccines for HIV-1 infection. Curr HIV Res (2003) 1(4):441–6. 10.2174/1570162033485168 15049429

[4] Sartorius R D’Apice L Trovato M Cuccaro F Costa V De Leo MG Antigen delivery by filamentous bacteriophage *fd* displaying an anti-DEC-205 single-chain variable fragment confers adjuvanticity by triggering a TLR9-mediated immune response. EMBO Mol Med (2015) 7(7):973–88. 10.15252/emmm.201404525 25888235 PMC4520660

[5] D’Apice L Costa V Sartorius R Trovato M Aprile M De Berardinis P. Stimulation of innate and adaptive immunity by using filamentous bacteriophage *fd* targeted to DEC-205. J Immunol Res (2015) 2015:585078. 10.1155/2015/585078 26380324 PMC4563097

[6] Parker ER Sethi A. Chagas disease: coming to a place near you. Dermatol Clin (2011) 29(1):53–62. 10.1016/j.det.2010.08.011 21095528

[7] Bern C Montgomery SP. An estimate of the burden of Chagas disease in the United States. Clin Infect Dis (2009) 49(5):e52–4. 10.1086/605091 19640226

[8] Bermudez J Davies C Simonazzi A Real JP Palma S. Current drug therapy and pharmaceutical challenges for Chagas disease. Acta Trop (2016) 156:1–16. 10.1016/j.actatropica.2015.12.017 26747009

[9] Boscardin SB Torrecilhas AC Manarin R Revelli S Rey EG Tonelli RR Chagas’ disease: an update on immune mechanisms and therapeutic strategies. J Cell Mol Med (2010) 14(6B):1373–84. 10.1111/j.1582-4934.2010.01007.x 20070438 PMC3829005

[10] Rodrigues MM Oliveira AC Bellio M. The immune response to *Trypanosoma cruzi*: role of toll-like receptors and perspectives for vaccine development. J Parasitol Res (2012) 2012:507874. 10.1155/2012/507874 22496959 PMC3306967

[11] Low HP Santos MA Wizel B Tarleton RL. Amastigote surface proteins of *Trypanosoma cruzi* are targets for CD8+ CTL. J Immunol (1998) 160(4):1817–23. 9469442

[12] Padilla A Xu D Martin D Tarleton R. Limited role for CD4+ T-cell help in the initial priming of *Trypanosoma cruzi*-specific CD8+ T cells. Infect Immun (2007) 75(1):231–5. 10.1128/IAI.01245-06 17043105 PMC1828400

[13] Aida Y Pabst MJ. Removal of endotoxin from protein solutions by phase separation using Triton X-114. J Immunol Methods (1990) 132(2):191–5. 10.1016/0022-1759(90)90029-U 2170533

[14] DaRocha WD Silva RA Bartholomeu DC Pires SF Freitas JM Macedo AM Expression of exogenous genes in *Trypanosoma cruzi*: improving vectors and electroporation protocols. Parasitol Res (2004) 92(2):113–20. 10.1007/s00436-003-1004-5 14634799

[15] Cummings KL Tarleton RL. Rapid quantitation of *Trypanosoma cruzi* in host tissue by real-time PCR. Mol Biochem Parasitol (2003) 129(1):53–9. 10.1016/S0166-6851(03)00093-8 12798506

[16] Helft J Bottcher J Chakravarty P Zelenay S Huotari J Schraml BU GM-CSF mouse bone marrow cultures comprise a heterogeneous population of CD11c(+)MHCII(+) macrophages and dendritic cells. Immunity (2015) 42(6):1197–211. 10.1016/j.immuni.2015.05.018 26084029

[17] Oliveira AC de Alencar BC Tzelepis F Klezewsky W da Silva RN Neves FS Impaired innate immunity in Tlr4(-/-) mice but preserved CD8+ T cell responses against *Trypanosoma cruzi* in Tlr4-, Tlr2-, Tlr9- or Myd88-deficient mice. PLoS Pathog (2010) 6(4):e1000870. 10.1371/journal.ppat.1000870 20442858 PMC2861687

[18] Vasconcelos JR Bruna-Romero O Araujo AF Dominguez MR Ersching J de Alencar BC Pathogen-induced proapoptotic phenotype and high CD95 (Fas) expression accompany a suboptimal CD8+ T-cell response: reversal by adenoviral vaccine. PLoS Pathog (2012) 8(5):e1002699. 10.1371/journal.ppat.1002699 22615561 PMC3355083

[19] Deng L Ibanez LI Van den Bossche V Roose K Youssef SA de Bruin A Protection against influenza A virus challenge with M2e-displaying filamentous *Escherichia coli* phages. PLoS One (2015) 10(5):e0126650. 10.1371/journal.pone.0126650 25973787 PMC4431709

[20] El-Sayed NM Myler PJ Bartholomeu DC Nilsson D Aggarwal G Tran AN The genome sequence of *Trypanosoma cruzi*, etiologic agent of Chagas disease. Science (2005) 309(5733):409–15. 10.1126/science.1112631 16020725

[21] Abrahamsohn IA Silva WD. Antibody dependent cell-mediated cytotoxicity against *Trypanosoma cruzi*. Parasitology (1977) 75(3):317–23. 10.1017/S0031182000051866 415286

[22] Umekita LF Takehara HA Mota I. Role of the antibody Fc in the immune clearance of *Trypanosoma cruzi*. Immunol Lett (1988) 17(1):85–9. 10.1016/0165-2478(88)90106-X 3127336

[23] Tzelepis F de Alencar BC Penido ML Gazzinelli RT Persechini PM Rodrigues MM. Distinct kinetics of effector CD8+ cytotoxic T cells after infection with *Trypanosoma cruzi* in naive or vaccinated mice. Infect Immun (2006) 74(4):2477–81. 10.1128/IAI.74.4.2477-2481.2006 16552083 PMC1418894

[24] Prisco A De Berardinis P. Filamentous bacteriophage fd as an antigen delivery system in vaccination. Int J Mol Sci (2012) 13(4):5179–94. 10.3390/ijms13045179 22606037 PMC3344273

[25] Reyes-Sandoval A Berthoud T Alder N Siani L Gilbert SC Nicosia A Prime-boost immunization with adenoviral and modified vaccinia virus Ankara vectors enhances the durability and polyfunctionality of protective malaria CD8+ T-cell responses. Infect Immun (2010) 78(1):145–53. 10.1128/IAI.00740-09 19858306 PMC2798185

[26] Betts MR Nason MC West SM De Rosa SC Migueles SA Abraham J HIV nonprogressors preferentially maintain highly functional HIV-specific CD8+ T cells. Blood (2006) 107(12):4781. 10.1182/blood-2005-12-4818 16467198 PMC1895811

[27] Almeida JR Price DA Papagno L Arkoub ZA Sauce D Bornstein E Superior control of HIV-1 replication by CD8+ T cells is reflected by their avidity, polyfunctionality, and clonal turnover. J Exp Med (2007) 204(10):2473. 10.1084/jem.20070784 17893201 PMC2118466

[28] Prezzemolo T Guggino G La Manna MP Di Liberto D Dieli F Caccamo N. Functional signatures of human CD4 and CD8 T cell responses to *Mycobacterium tuberculosis*. Front Immunol (2014) 5:180. 10.3389/fimmu.2014.00180 24795723 PMC4001014

[29] Dc-Rubin SS Schenkman S. *Trypanosoma cruzi* trans-sialidase as a multifunctional enzyme in Chagas’ disease. Cell Microbiol (2012) 14:1522–30. 10.1111/j.1462-5822.2012.01831.x 22747789

[30] Nardy AF Freire-de-Lima CG Perez AR Morrot A. Role of *Trypanosoma cruzi* trans-sialidase on the escape from host immune surveillance. Front Microbiol (2016) 7:348. 10.3389/fmicb.2016.00348 27047464 PMC4804232

