# Supplementary material for: Vaccination With Recombinant Filamentous fd Phages Against Parasite Infection Requires TLR9 Expression
Source: Front Immunol. 2018 May 29;9:1173. doi: 10.3389/fimmu.2018.01173 (PMC5987186; doi:10.3389/fimmu.2018.01173)
Supplement: Supplementary file 1 [file data_sheet_1.PDF]

Vaccination with recombinant filamentous *fd* phages against parasite infection requires TLR9 expression.

João F. Gomes-Neto[1], Rossella Sartorius[2], Fábio B. Canto[1], Thamyres S. Almeida[1], André A. Dias[1], Carlos-Henrique D. Barbosa[1], Guilherme A. Melo[1], Ana Carolina Oliveira[3], Pedro-Henrique N. Aguiar[4], Carlos R. Machado[4], Herbert M. Guedes[5], Marcelo F. Santiago[3], Alberto Nóbrega[1], Piergiuseppe De Berardinis[2] and Maria Bellio[1,6]*

Supplementary Figures and Table

Supplementary Figure 1

A

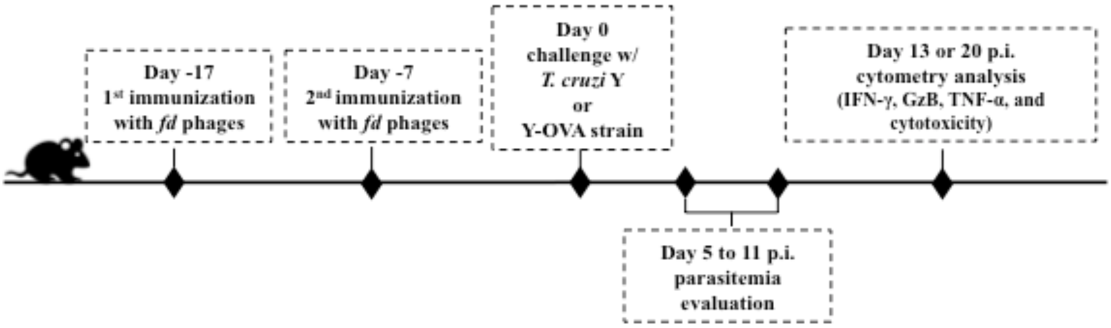

B

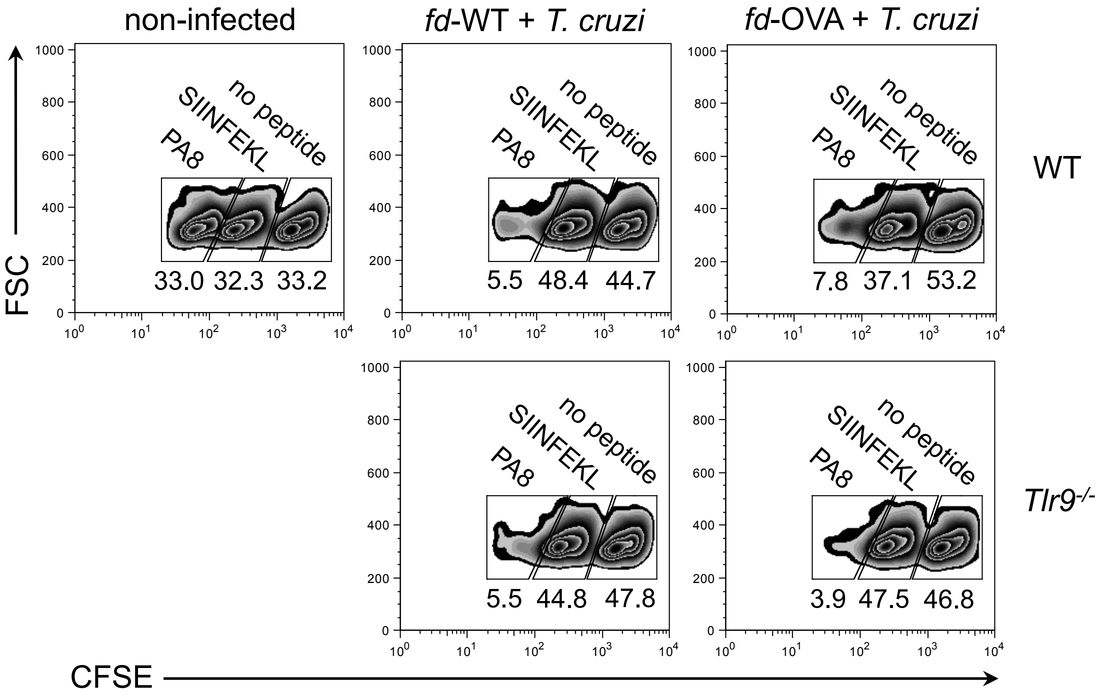

Supp. Figure 1: ***Immunization scheme and representative contour plot of in vivo cytotoxicity assay:*** Mice were injected ip with 100 μg of *fd* phages at day -17 and -7 and infected with *T.cruzi* Y or Y-OVA trypomastigotes at day 0. Sera employed in ELISA assays were collected on day 8 or 13 pi. In the experiment shown in Figure 6, *in vivo* cytotoxicity was analyzed on day 8 and 106 pi. **(A)**. Representative contour plot of the *in* vivo cytotoxicity assay shown on Figure 1E **(B).**

Supplementary Figure 2

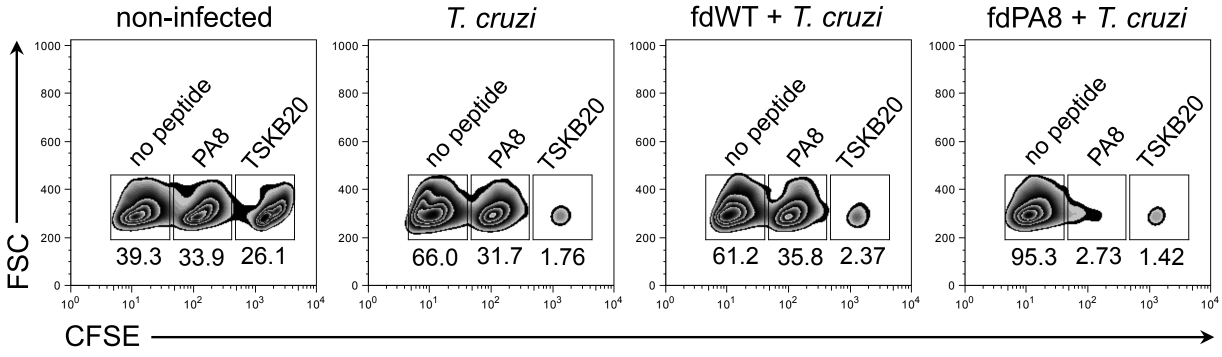

Supp. Figure 2: ***Representative contour plot of in vivo cytotoxicity assay:*** Representative contour plot of the *in* vivo cytotoxicity assay shown on Figure 2B.

Supplementary Figure 3

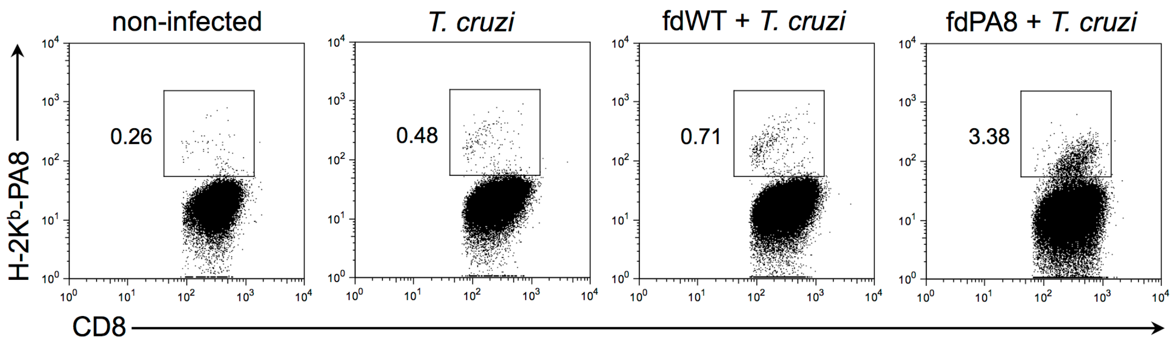

Supp. Figure 3: ***Representative dot plot of staining with H-2K^b-PA8 pentamer:*** Representative dot plot of the staining with H-2K^b-PA8 pentamer shown on Figures 3A and 3E.

Supplementary Figure 4

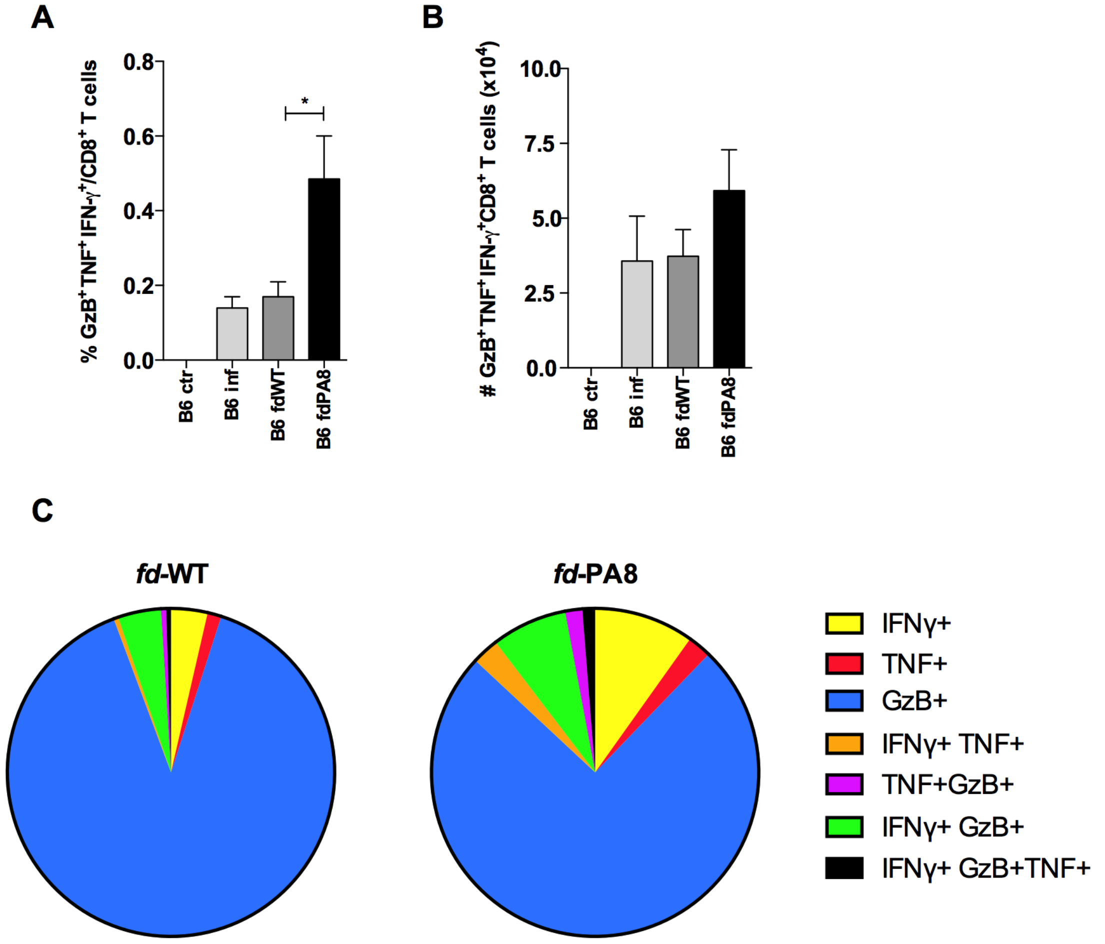

Supp. Figure 4: ***Immunization with fd-PA8 phages increases the levels of polyfunctional CD8$^+$ T cells in the spleen of B6 mice.*** Male mice were immunized (as in Supp Fig.1) and infected on day 0 with 2 x 10^3 blood trypomastigotes of the Y strain. On Day 13 pi, splenocytes from control naïve (white bars), infected-only (light grey bars), *fd*-WT (dark grey bars) or *fd*-PA8 immunized mice (black bars) were stained following a 10-h *in vitro* incubation with PA8 peptide, as described on the Material and Method section. Mean percentages **(A)** and absolute numbers **(B)** of GzB$^+$TNF$^+$IFN-γ$^+$ CD8$^+$ T cells (triple positive CD8$^+$ T cells) of individually analyzed mice (n=5) are shown. Chart graphics **(C)** representing distribution of the single and multiple cytokine/GzB producing CD8$^+$ T cells in the spleen of *fd*-WT- and *fd*-PA8-immunized and infected mouse groups shown in (A), (B) and in Figures 3 and 4. Error bars= SEM, *p≤ 0.05 (two-tailed Student t test).

Supplementary Table 1.

Presence of the PA8 epitope (VNHRFTLV) in other proteins, members of the *T. cruzi* *trans*-sialidase super-family.

**Hit #**	**NCBI Genbank Access number**	**Size**	**% Identity**
1	XP_803984.1	794 aa	100%
2	XP_810614.1	728 aa	100%
3	XP_804688.1	704 aa	100%
4	XP_814809.1	239 aa	100%
5	XP_812580.1	213 aa	100%
6	XP_802177.1	210 aa	100%

All results were obtained by BLAST search at 100 % coverage
